# Optical Interferometric Fringe Pattern-Incorporated Spectrum Calibration Technique for Enhanced Sensitivity of Spectral Domain Optical Coherence Tomography

**DOI:** 10.3390/s20072067

**Published:** 2020-04-07

**Authors:** Sangyeob Han, Ruchire Eranga Wijesinghe, Deokmin Jeon, Youngmin Han, Jaeyul Lee, Junsoo Lee, Hosung Jo, Dong-Eun Lee, Mansik Jeon, Jeehyun Kim

**Affiliations:** 1 School of Electronics Engineering, College of IT Engineering, Kyungpook National University, 80, Daehak-ro, Buk-gu, Daegu 41566, Korea; syhan850224@knu.ac.kr (S.H.); dmjeon@knu.ac.kr (D.J.); jaeyul@knu.ac.kr (J.L.); jslee5399@knu.ac.kr (J.L.); ghtjd_419@naver.com (H.J.); jeehk@knu.ac.kr (J.K.); 2 Department of Biomedical Engineering, College of Engineering, Kyungil University, 50, Gamasil-gil, Hayang-eup, Gyeongsan-si, Gyeongsangbuk-do 38428, Korea; eranga@kiu.kr; 3 Department of Autonomous Robot Engineering, College of Smart Engineering, Kyungil University, 50, Gamasil-gil, Hayang-eup, Gyeongsan-si, Gyeongsangbuk-do 38428, Korea; 4 Department of Nuclear Energy Convergence, Kyungil University, 50, Gamasil-gil, Hayang-eup, Gyeongsan-si, Gyeongsangbuk-do 38428, Korea; anym@kiu.kr; 5 School of Architecture and Civil Engineering, Kyungpook National University, 80, Daehak-ro, Buk-gu, Daegu 41566, Korea

**Keywords:** optical coherence tomography (OCT), Point Spread Function (PSF), depth visualizing sensitivity, spectral calibration, SD-OCT

## Abstract

Depth-visualizing sensitivity can be degraded due to imperfect optical alignment and non-equidistant distribution of optical signals in the pixel array, which requires a measurement of the re-sampling process. To enhance this depth-visualizing sensitivity, reference and sample arm-channeled spectra corresponding to different depths using mirrors were obtained to calibrate the spectrum sampling prior to Fourier transformation. During the process, eight interferogram patterns corresponding to point spread function (PSF) signals at eight optical path length differences were acquired. To calibrate the spectrum, generated intensity points of the original interferogram were re-indexed towards a maximum intensity range, and these interferogram re-indexing points were employed to generate a new lookup table. The entire software-based process consists of eight consecutive steps. Experimental results revealed that the proposed method can achieve images with a high depth-visualizing sensitivity. Furthermore, the results validate the proposed method as a rapidly performable spectral calibration technique, and the real-time images acquired using our technique confirm the simplicity and applicability of the method to existing optical coherence tomography (OCT) systems. The sensitivity roll-off prior to the spectral calibration was measured as 28 dB and it was halved after the calibration process.

## 1. Introduction

Optical coherence tomography (OCT) enables micrometer-resolution biomedical imaging to be performed in real time [1,2,3]. Due to its capability to perform non-invasive inspection, OCT has been extensively utilized and featured in numerous scientific research and clinical studies [4,5,6,7,8,9,10]. In addition to its medical applications, spectral domain OCT (SD-OCT) based field inspections have also been applied to industrial and agricultural materials [11,12,13,14,15,16]. One issue with SD-OCT, however, is that the sensitivity of its depth-dependent signals tend to be weaker in deeper imaging regions [17,18,19], as a cause of slight technical errors associated with the optical spectrometer.

An optical spectrometer should, therefore, be designed according to a specific center wavelength and a full width at half maximum (FWHM) bandwidth; this will allow it to obtain wavenumber measurements that are uniformly distributed among an array of pixels. If the interference fringe signal of a SD-OCT is subjected to a discrete Fourier transformation, then its performance can be optimized once the Fourier transforming data points are equally distributed [20]. The Fourier transformation operates in the frequency domain, while the camera pixel array produces a linear representation of the spectrum in terms of its wavelength, not frequency. In OCT, the final image is a convolution of the system point spread function (PSF) and the object being imaged. Therefore, an object that is a delta function would provide a direct measurement of the point-spread limited resolution [21]. If the data are not linearized by being inserted into equidistant frequency slots, the depth-dependent PSF can be distorted, and each pixel of the line-scan camera of the optical spectrometer can then integrate with a different spectral width [22,23,24]. Spectral calibration methods have, therefore, always been a primary concern. Numerous approaches were demonstrated by using complex numerical data resampling methods and using bulky optical hardware methods due to unavailability of supremely well-designed spectrometers [25,26,27,28,29]. 

Resampling schemes involving a graphics processing unit (GPU) have been frequently proposed in relevant studies to convert the wavelength domain to the wavenumber domain enhancing the data acquisition speed [20,30]. In SD-OCT, a technique for linearization of spectral fringe signals has been demonstrated by correcting the ν = c/λ relation with both software and optical hardware elements by extracting accumulative phase evolution of the interference fringes [30,31,32,33]. In the aforementioned techniques, the interference fringe patterns were processed using linear interpolation with fast Fourier transformation (FFT), cubic spline interpolation with FFT, non-uniform discrete Fourier transforms (NDFT), and non-uniform fast Fourier transforms (NFFT). Furthermore, compressed sensing methods that are based on wavenumber domain linearizing techniques have been demonstrated to simplify complex numerical methods [18,26]. Some studies have also focused on optical hardware optimizations by using a wavelength-scanning filter technique, which provides lookup tables instead of interpolating non-linear data [34,35]; other studies have used prism implementations to achieve real-time imaging [17,25,36]. 

Simultaneous correction of non-linear *k*-sampling and dispersion mismatch between sample and reference arms was demonstrated by extracting two calibration vectors for the numerical resampling for wavenumber-linearization and phase correction for the dispersion mismatch [37]. Multiple complex numerical steps with linear and non-linear terms were assessed to determine the position of the peak in A-lines and peak broadening distortion, respectively. Additionally, a full-range visualizing technique was integrated along with spectral and dispersion calibration, which enhances the OCT providing a large depth range [38]. To eliminate the necessity of linearization, a dual interferometer-based (master and slave interferometers) approach was developed to operate with a sensitivity similar to SD-OCT providing signal from a single point in depth [39,40] and a similarly complex master slave OCT method was performed to process the interferometer spectrum without Fourier transformation. Semi-automatic spectral mapping is another technique, which was acquired using dual spectrometers, where a set of calibration interference fringes has to be acquired from both spectrometers [41,42]. In these two aforementioned methods [41,42], non-linearity in the unwrapped phase information was sampled by generating linear vectors. For these newly generated linear vectors, a fractional index through interpolation corresponding to its position in the original phase vector can be identified. Thereafter, these fractional indices can be used to resample all subsequent measurements in order to obtain wavenumber linearization samplings. Once wavenumber linearization is completed, A-lines of the interference signal are acquired to find peak axial positions followed by Gaussian filtering, fast Fourier transformation, and further signal processing [37]. 

In this paper, we present a simply implementable 8-step alternative spectral calibration method to overcome the major drawback of SD-OCT imaging systems. The method only requires raw data of optical PSF resolved interferograms, which is then fed into a customized computational algorithm. The SD-OCT hybrid algorithm encapsulates binarization, intensity detection, interpolation, and indexation, which are performed in real-time. The acquired raw data of multiple adjacent depth positions were well utilized in the developed algorithm to simply functionalize for any broadband laser and line scan camera of SD-OCT. Therefore, the method proposed here can be easily integrated into current SD-OCT systems to overcome spectral broadening limitations and to obtain an enhanced depth-dependent sensitivity fall-off.

## 2. Materials and Methods 

### 2.1. Description of Optical Coherence Tomography (OCT) System Configuration

**Figure 1:**
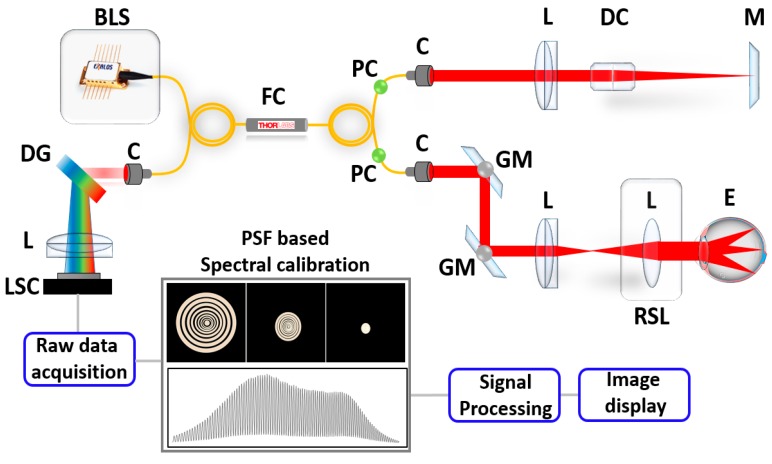
System configuration of the SD-OCT system. Abbreviations: BLS, broadband laser source; C, collimator; DG, diffraction grating; DC, dispersion compensator; E, eye; FC, fiber coupler; GM, Galvano mirror; L, lens; LSC, line scan camera; M, mirror; PC, polarization controller; RSL, retinal scanning lens.

Built upon spectral interferometry, SD-OCT uses an optical interference signal consisting of a sample arm beam and a reference arm beam, with an exactly-matched optical path length difference. The experimental setup of our study featured a retinal inspection OCT (shown in Figure 1) that was developed for the direct implementation of the proposed scheme. The system was operated with a broadband light source (EXS210022-02, Exalos, Switzerland) that had a center wavelength of 840 nm and a bandwidth of 50 nm. A 50:50 optical fiber coupler (TW850R5A2, Thorlabs, Newton, NJ, USA) was used to split the optical light beams into a sample arm and a reference arm, and the backscattered optical light beams were recorded by a laboratory-customized spectrometer, containing a diffraction grating with 1800 lines/mm and a line scanning camera (2048 pixels, E2V, UK). During image acquisition, 500 B-scans were acquired in 6 seconds. Each B-scan consisted of 500 A-scans. The detailed optical configurations and system specifications of the retinal OCT instrumentation including retinal scanning lens configuration have been previously reported elsewhere [43].

### 2.2. Spectral Calibration Algorithm

**Figure 2:**
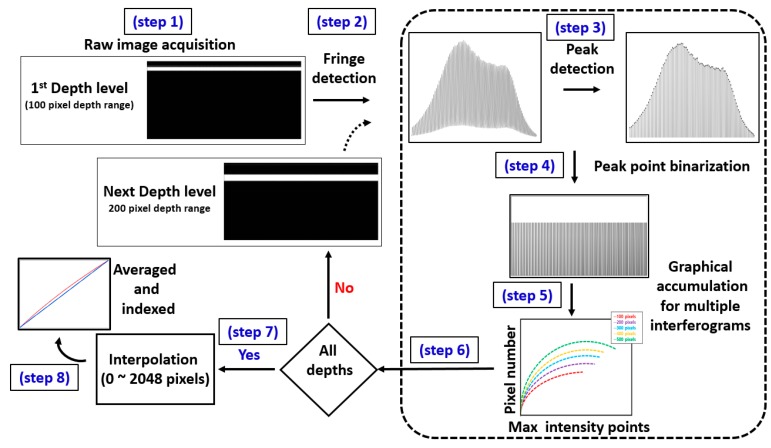
Graphical representation of the fundamental steps of the software-based intensity detection and resampling algorithm developed for the spectral calibration.

Figure 2 illustrates the 8 simple fundamental steps of the software-based intensity detection and resampling algorithm, developed for spectral calibration. We obtained PSF incorporated spectrums by using two mirrors as reference and sample arms, starting from 100 pixel depth range, towards 800 pixel depth range, at eight optical path length differences (shown in step one). Step two emphasized the extraction of the interferogram pattern using PSF, which was utilized as baseline data. We then applied an intensity-based peak detection filter to each interferogram to select the maximum intensity points (shown in step 3) and the selected points were later binarized (shown in step 4). Then, the pixel position corresponding to these each binarized maximum intensity point was graphically rearranged and plotted as pixel number (position) against maximum intensity point (shown in step 5). An analogous procedure was repeated for the remaining seven depth levels (200 pixel level to 800 pixel depth range) and the program executed continuously until the condition of step 6 was satisfied. Thus, the algorithm repetitively executed for eight PSF trials. The spectral graphs including spectrally encoded multiple interference fringes from multiple depths were streamlined to enhance the imaging depth and signal-to-noise ratio (SNR) [32]. When PSF-based data acquisition was completed across all of the depth levels, the algorithm executed step 7, which was the cubic spline interpolation [44] step of the maximum intensity points. As the number of maximum intensity points of each depth level was dissimilar, all intensity points had to be interpolated to the total number of pixels of the OCT line scan camera (total number of pixels in current study: 2048). Therefore, during this step 7, the maximum intensity points were resampled using nearest adjacent intensity points coinciding with sampled data at the interpolated nodes. Once this interpolation was completed, the dimensions of each depth level pixel array were similar to each other. In the current experiment, eight PSF trials were performed and therefore, eight curves illustrating correlated pixel numbers (pixel positions) at each number of points with maximum intensity were arranged, summed up, and averaged to generate a single representative curve (shown in step 8). Next, a linear curve was sampled with similar number of data samples and plotted along with the interpolated data, which was linearly fitted later through indexing the non-linear sample points. Finally, a lookup table was generated based on these linearizing index values to rescale real-time raw data enhancing the OCT image. Since the key baseline data required for the developed algorithm operation was the extracted intensity information of PSF based interferogram patterns, the algorithm could be functionalized well despite the FWHM bandwidth of optical broadband laser sources (≥50 nm (FWHM)). 

**Figure 3:**
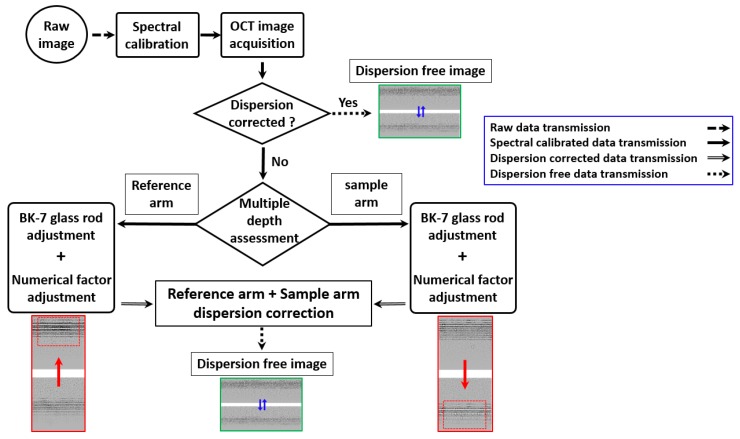
Flow diagram of the fundamental steps performed for dispersion of the system. The red color solid arrows illustrate switching image states (changing of images), while compensating dispersion using decoupled hardware and software methods. The blue color solid arrows illustrate switching image states, while changing the focusing position.

Moreover, cubic-spline interpolation is a complex numerical calibration technique, where the image pixels are stored in a compute unified device architecture (CUDA) array and bound to a designated region in a texture memory. Resampled data points are generated by interpolating the raw fringe data points, which eventually provides evenly distributed pixel information. From the entire array of pixels, raw data were extracted from 0~800 pixel depth range due to weakened fringe information (less intensity) of the extreme corner beyond the 800 pixel range. Therefore, 8 PSF measurements (n = 8) were acquired with a pixel interval of 100 pixels, which provided sufficient raw data of the entire depth range with least missing depth information that eventually led to a precise spectral calibration. Fewer PSF measurements (n < 8) will reduce the preciseness of data with lack of depth information, which has negative impact on distinguishing deep layers of OCT B-scans. Although non-uniform data point distribution was corrected, a slight dispersion was identified on the image due to mismatch between the reference and sample propagating paths, where the light is specularly reflecting from the mirror surface; and secondly, due to depth-dependent dispersion of the sample with several layers of coverslip glass. Thus primarily, the spectral calibration was performed to enhance the depth dependent sensitivity, and secondly the dispersion mismatch was compensated using both hardware (dispersion compensating glass-rods) and numerical operations based software methods. Figure 3 illustrates the flow diagram of the fundamental steps performed after the spectral calibration to further compensate dispersion of the system. To decouple two technical contributions (spectral calibration and dispersion compensation), a single frequency channeled spectrum with no dispersion compensation was acquired in the original state of the image initially. This data stream contains different sampling information between sample and reference arm, which is the main cause for the image dispersion. Next to confirm the exact optical arm with dispersion, BK-7 glass rods (dispersion compensating glass rods) were placed on reference arm. A sample consists of multiple (five) glass cover slips representing multiple depth levels was placed on sample arm and the image was visualized. Raw data of the corresponding image was extracted and arranged to examine dispersion variation. This set of raw data was arranged and inserted to the spectrum calibration algorithm. Instead of using the phase information of the interferogram, fundamental index intensity of the interferogram was varied to compensate dispersion. A numerical multiplication factor with an intensity weight (numerical weight with hundredth decimal point starting from 1) was utilized in the algorithm to match the dispersion of all the corresponding depth levels. The utilization of this numerical multiplication factor results in adjustments of the index weight and sampling of the entire array, which interpolates an index to calibrate the spectrum. The exact numerical factor, which calibrates the spectrum is verified after successful interpolation attempts, and the entire interpolated raw index array is Fourier transformed and log scaled for the image visualization. Simultaneously, the index intensity can be continuously varied to enhance the image quality until the spectrum calibration is satisfactorily accomplished. The performed BK-7 based reference arm dispersion compensation can be successfully confirmed if the acquired image reveals a similar thickness in all depth levels of the sample. When a thickness difference is identified at deeper regions, dispersion of the sample arm has to be corrected using additional BK-7 glass rods until a sufficient dispersion compensation with similar thickness for all depth levels is obtained. Subsequently, a new set of raw data can be arranged and index intensity has to be varied through the numerical factor of the spectrum calibration algorithm to match further dispersion of the entire depth range. The utilization of this numerical factor results in adjustments of the index weight and sampling of the entire array, which simply leads to an index interpolation to compensate dispersion. As a result of this decoupled operation of the spectrum calibration technique and dispersion compensation technique, the dispersion mismatch between reference and sample arms can be compensated by cancelling the frequency-dependent real and the imaginary parts of the spectral fringe pattern, which are used to construct the complex analytical representation of the spectral fringe pattern.

### 2.3. In Vivo Experimental Procedure

We performed in vivo SD-OCT imaging of the human retina with and without adoption of the developed spectral calibration technique. All human experiments were performed in accordance with the guidelines of the Institutional Animal and Human Care and Use Committee of Kyungpook National University (No. KNU-2018-0100). We captured two image sets, each consists of pair of two dimensional OCT (2D-OCT) images of an exact location of human retina. We further utilized our developed spectral calibration method to confirm the progressive OCT image quality improvement by examining in vivo Sprague Dawley rat posterior chambers. The preparation of the Sprague Dawley rat specimens and the anesthetic procedure were performed according to a previously reported method [45]. The in vivo rat eye specimens were repeatedly scanned to obtain a three-dimensional (3D) enface visualization of their posterior vitreous opacifications. The calibrated volumetric OCT images were averaged for speckle noise reduction and cross-sections were extracted corresponding to four particular positions (position 1–position 4). In addition to the retinal posterior vitreous assessments, in vivo anterior vitreous visualizations were obtained also from in vivo Sprague Dawley rat specimens. B-scans were obtained before and after performing the developed spectral calibration technique. Unlike posterior segment imaging systems, anterior segment scanners require illumination of the eye with a focused optical beam. Therefore, the retinal scanning lens (RSC) of schematic 1 (Figure 1) can be removed temporarily to fulfill this purpose.

## 3. Results and Discussion

### 3.1. Analysis of Point Spread Function (PSF) Incorporated Spectral Calibration

**Figure 4:**
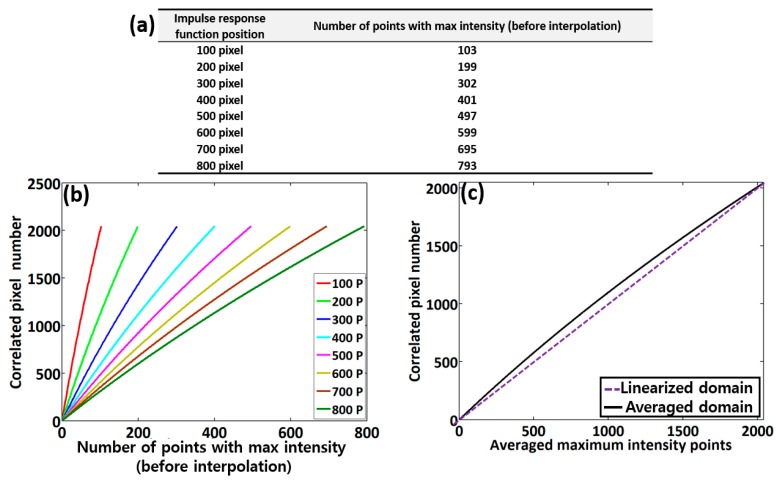
Evaluations of the point spread function (PSF)-based spectral interferogram analysis. (**a**) The depth information table of each PSF position, showing the respective number of points carrying maximum intensity, (**b**) correlated pixel numbers (pixel positions) at each number of points with maximum intensity prior to interpolation, and (**c**) the averaged graphical plot of the eight respective graphs in Figure 4b.

The initial experimental procedure of the proposed scheme is the acquisition of spectral interferograms corresponds to multiple PSF trials. The table illustrated in Figure 4a provides depth information for each PSF position starting from the 100th-pixel to 800th-pixel, along with the number of points carrying the maximum intensity in each corresponding interferogram. Since a non-calibrated spectrometer broadens the spectral width along with the depth of the PSF, this broadening effect increases the number of points carrying the maximum intensity. To overcome this dissimilarity, we interpolated each set of maximum intensity points, and converted them to 2048 (the number of pixels in the OCT camera pixel array). 

Figure 4b shows the correlated pixel numbers (pixel positions) at each number of points with maximum intensity prior to interpolation acquired during the assessment of each depth-dependent PSF position. The solid black color curve of Figure 4c emphasizes the summated and averaged curve of eight corresponding curves of Figure 4b illustrating pixel position information over each averaged maximum intensity point. From the results, it is seen that the practical point distribution is no longer linear owing to the spectral errors that occur in spectrometer. Therefore, we deliberately generated a calibrated domain, where the non-linear points were re-indexed and linearized. The purple dashed curve shows the deliberately generated linear domain obtained after curve fitting, which is initiated from the lowest averaged maximum intensity point to uppermost point. We used the closest method for a linear pattern by selecting the closest value between the measured averaged intensity point and the linear point, where the index values were later utilized in a real-time image acquisition program.

### 3.2. Depth-Dependent Sensitivity Assessments after Spectral Calibration

**Figure 5:**
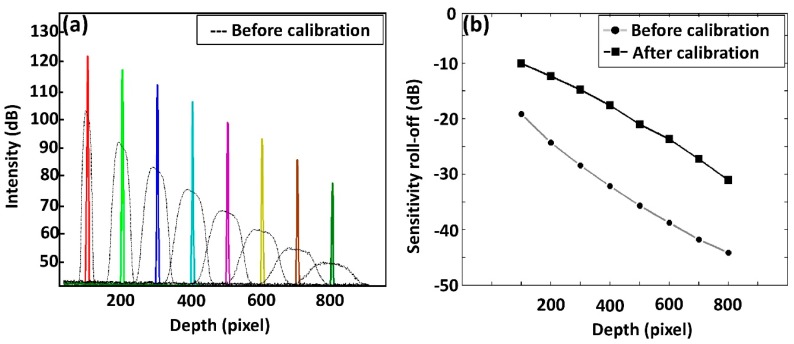
Quantitative representation of system sensitivity assessments. (**a**) Depth-dependent system intensity fall-off and (**b**) depth-dependent sensitivity roll-off (signal-to-noise ratio, SNR) representation. Each PSF peak of Figure 4a corresponds to the correlated pixel numbers of Figure 4b.

Figure 5a shows the depth dependent intensity fall-off of the system, where we obtained PSFs before and after applying the spectral calibration technique. The black dashed curve shows the intensity roll-off before calibration verifying the broadening effect of the spectral width. The multicolor solid sharp peaks show the intensity roll-off after the spectrum was calibrated. The comparison shown in Figure 5a clearly reveals that the averaged fall-off curve acquired after the spectral calibration exhibits much less decay than the averaged non-calibrated roll-off, across the whole imaging depth range. Similarly, the depth-dependent sensitivity roll-off emphasizing signal-to-noise ratio (SNR) graphs acquired before and after spectral calibration are plotted in Figure 5b. A significant sensitivity roll-off (28 dB) occurs prior to the spectral calibration, while the intensity fall-off was 14 dB after calibration.

### 3.3. Full Width at Half Maximum (FWHM) Evaluation of Depth-Dependent PSF

**Figure 6:**
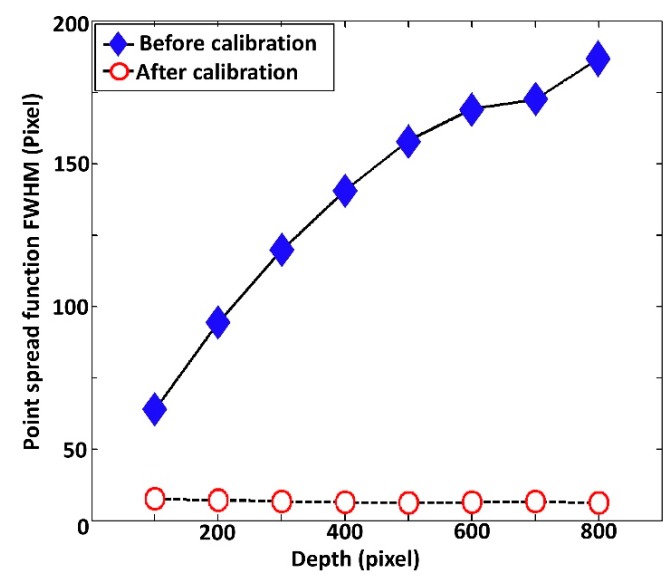
Full width at half maximum (FWHM) comparison between before (without) and after (with) spectral calibrated depth-dependent PSFs.

We further examined and compared the FWHM between before and after spectral calibrated depth-dependent PSFs as shown in Figure 6. Our primary objective of this evaluation was to gain a better understanding of the depth visualizing sensitivity and pixel-resolution of deep structures, as the FWHM has a direct impact on the pixel-resolution incorporated axial resolution. Thus, the evaluated FWHM of the peaks are presented as pixel units, which have a direct relationship with thickness. According to the graphical representation, Figure 6 suggests that the trend of the non-calibrated PSF width increasingly fluctuates in the deeper regions, while the width of the spectrally-calibrated PSF remains relatively constant across the whole imaging depth range. Henceforth, this comparative evaluation demonstrates that the developed spectral calibration method effectively improves depth-visualizing sensitivity and resolution in deeper imaging regions for SD-OCT systems. Therefore, examined depth-dependent PSF intensity fluctuation and sensitivity roll-off of our method emphasized a higher sensitivity with less noise residual confirming the potential merits over previously reported conventional techniques [17,26].

### 3.4. Implementation of Dispersion Correction 

**Figure 7:**
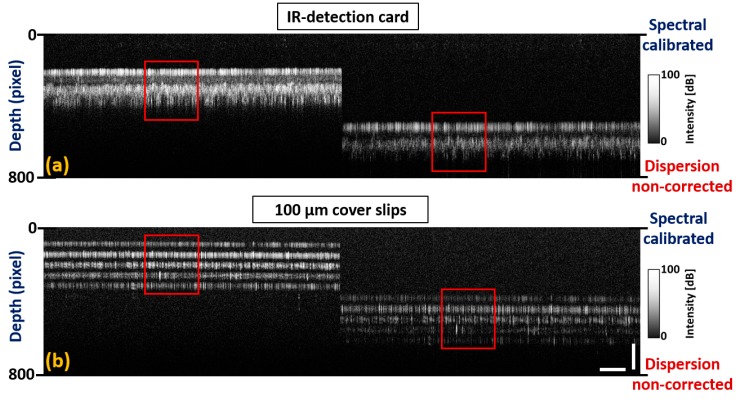
Spectrum calibrated two dimensional OCT images acquired prior to dispersion compensation. (**a**) 2D-OCT images of the infrared (IR)-detection card at top and bottom depth levels. (**b**) 2D-OCT images of five glass cover slips at top and bottom depth levels. Red color square boxes represent a region of interest with non-corrected dispersion. Horizontal scale bar: 1 mm, vertical scale bar: 300 μm.

After performing a spectral calibration process, initial examination of the developed spectrum optimization was assessed using infrared (IR) detection card and a set of five glass cover slips. Figure 7a,b depict 2D-OCT images of the IR-detection card and set of five glass cover slips acquired after spectrum calibration.

**Figure 8:**
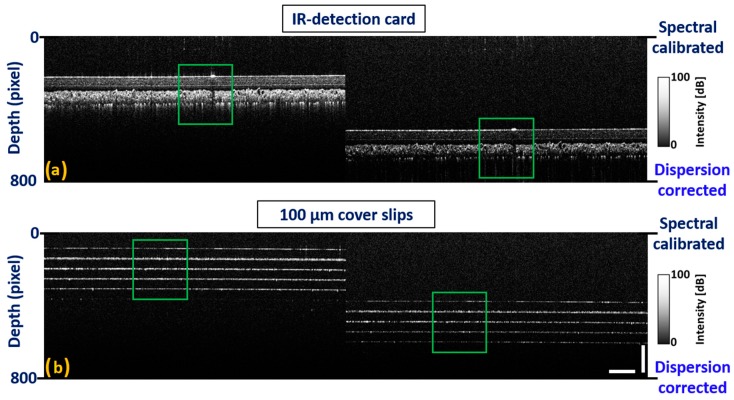
Spectrum calibrated two dimensional OCT images acquired after performing dispersion compensation. (**a**) 2D-OCT images of IR-detection card at top and bottom depth levels. (**b**) 2D-OCT images of five glass cover slips at top and bottom depth levels. Green color square boxes represent a region of interest with corrected dispersion. Horizontal scale bar: 1 mm, vertical scale bar: 300 μm.

Although the individual layer thickness of both samples at the top depth level was similarly distributed at the bottom depth level, sub-surface structural properties were not identified distinguishably due to a dispersion mismatch between the sample and reference arms. BK-7 glass rods were placed on both reference and sample arms, and the number of rods was interchanged until a certain amount of dispersion was compensated for. To overcome the remaining dispersion mismatch between two arms, the fundamental index intensity of the raw index of interference signal was varied using a numerical multiplication factor with hundredth decimal point. The acquired spectral calibration and dispersion correction can be confirmed in Figure 8a,b. The results reveal that the layer thickness in both samples was similarly distributed at both depth levels along with significantly distinguishable sub-surface structural properties. 

### 3.5. In Vivo OCT Image Verification of the Developed Spectral Calibration Method

**Figure 9:**
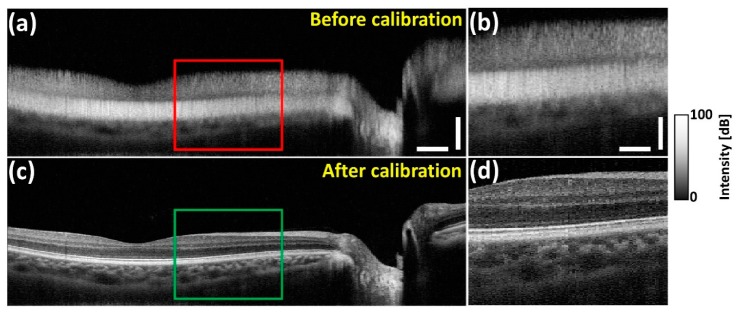
Image comparison with and without spectral calibrated in vivo human retina. (**a**) In vivo human retinal image before (without) spectral calibration; (**b**) shows magnified view of red box. (**c**) In vivo human retinal image after (with) spectral calibration; (**d**) shows magnified view of green box. (**a**) Horizontal scale bar: 1 mm, vertical scale bar: 300 μm, (**b**) horizontal scale bar: 500 μm, vertical scale bar: 150 μm.

In Figure 9, two image sets, each consists of pair of 2D-OCT images of an exact similar location of the human retina are presented. Figure 9a illustrates the 2D-OCT image acquired prior to the calibration and Figure 9c shows the image acquired after employing the calibration. Figure 9b,d are the zoomed images of the region of interest indicated on Figure 9a,c. Noticeably, the 2D-OCT image shown in Figure 9a emphasizes a less depth-visualizing sensitivity and less resolution in deep structures (in the case where no spectral calibration was performed) compared to Figure 9c, where the qualitative representation was significantly enhanced after calibration. Thus, in terms of SD-OCT based future clinical imaging applications, the developed technique shows good stability and reproducibility for any type of SD-OCT system.

**Figure 10:**
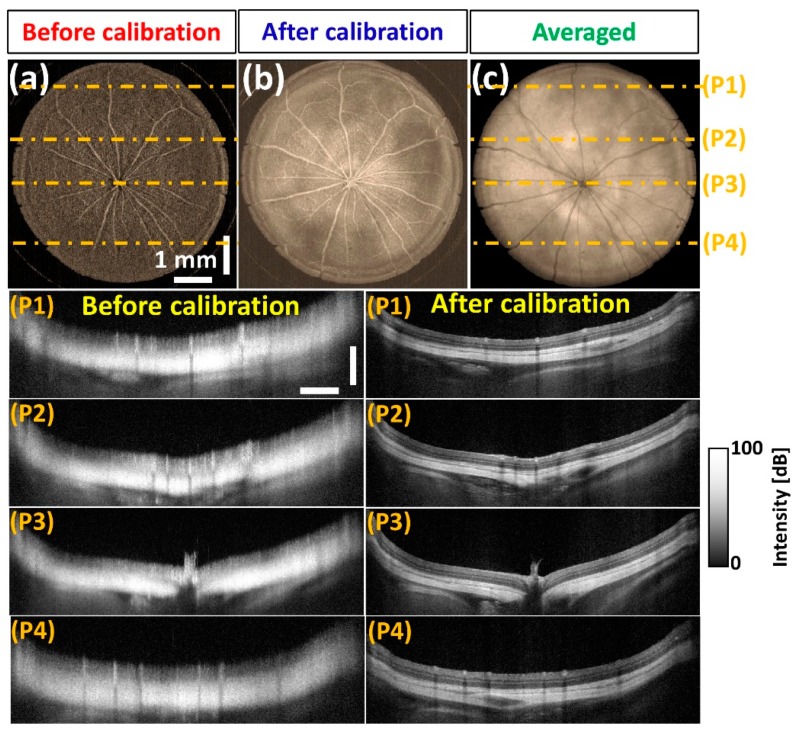
OCT image verification of *in vivo* rat posterior chamber along with B-scans corresponding to multiple positions. (**a**) Before calibration, (**b**) after calibration, (**c**) averaged posterior vitreous opacifications. The horizontal and vertical scale bars of P1–P4 (position 1–position 4) are 1 mm and 700 μm, respectively.

The developed spectral calibration method was further utilized to confirm the progressive OCT image quality improvement by examining in vivo Sprague Dawley rat posterior vitreous opacifications. Figure 10a,b exhibit structural differences and image enhancements between the images taken before and after the calibration process revealing a higher visibility of blood vessels in 10b. The calibrated 3D-OCT images were further averaged for speckle noise reduction (Figure 10c) and B-scans corresponding to four particular positions (p1~p4) were extracted from both Figure 10a,c as indicated in Figure 10.

**Figure 11:**
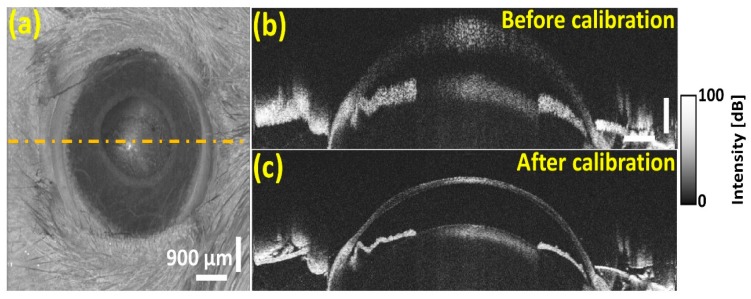
The OCT image verification of *in vivo* rat anterior vitreous opacification along with B-scans corresponds to multiple positions. (**a**) 3D enface visualization of anterior vitreous opacification, (**b**) before calibration, and (**c**) after calibration. The horizontal and vertical scale bars of (**b**) are 500 μm and 300 μm, respectively.

Figure 11a depicts the enhanced 3D-OCT enface visualization of the anterior vitreous opacification, along with B-scans that were obtained before and after performing the spectral calibration technique. In particular, the effective depth visualizing sensitivity enhancement of the anterior segment including cornea, iris, and lens region can be clearly observed after the utilization of our spectral calibration technique. Hence, the results of Section 3.4 qualitatively verified that the developed calibration method performed over the entire spectrum remarkably enhanced the visualization of microstructural features of transparent mediums, such as anterior and posterior vitreous owing to the enhanced depth-visualizing sensitivity and image resolution of deep structures. 

Similar to previously reported methods [37,38,39,40,41,42], the developed fringe pattern-incorporated spectrum calibration method is applicable to enhance the sensitivity of the SD-OCT system and to compensate the dispersion mismatch as a simultaneous approach. Indeed, it relies on intensity information of the interferometric fringe signals, and the adjustable intensity weight (multiplication factor) confirmed the simple functionality and lower complexity of the proposed method compared to conventional methods. Although the current approach is closely related to the methods reported in [37,38,39] in the introduction, rapid calibration of the spectrum along with dispersion compensation using a single inferometer (without any other complex hardware) is still an important difference. Moreover, the approach described in this manuscript minimized the necessity of complex numerical calculations, which dramatically reduces computational time. Since the developed method is particularly relevant for SD-OCT systems consisting of broadband lasers with any center wavelength at any FWHM bandwidth and capable of real-time rectification of spectrum non-linarites and dispersion mismatch, extended ophthalmological applications can be anticipated.

## 4. Conclusions

In this study, we introduce an optical PSF resolved interferogram-incorporated spectral calibration technique as an uncomplicated alternative technique to overcome the depth-dependent sensitivity loss commonly experienced in SD-OCT systems. The validation of a comprehensive 8 step-based spectral calibration technique was applied for in vivo human retinal imaging and in vivo retinal and corneal imaging of Sprague Dawley rats. The experimental results, including quantifications and qualitative in vivo assessments, sufficiently confirmed the simplicity and accuracy of our technique along with an axial resolution of 8 μm (measured in air) and maximum imaging depth of 2.6 mm (in air). Moreover, the comparison between non-calibrated and calibrated spectral information revealed its methodological precision, and highlighted that it was able to decrease unfavorable sensitivity fall-off and significantly increase depth-visualizing sensitivity and image resolution in deep cross-sectional regions. Therefore, this calibration method is suitable to use with any standard SD-OCT system, as a simple incorporative spectral calibration technique.
